# Unraveling the impact of pilot fuel injection pressure on hydrogen-diesel engine performance through PCA and RSM analysis

**DOI:** 10.1038/s41598-026-39923-4

**Published:** 2026-03-02

**Authors:** Avadhoot A Mohite, Nitin Kumar, Debasis De, Bhaskor J Bora, Bhaskar J Medhi, Prabhu Paramasivam, Mohamed Yusuf

**Affiliations:** 1 Rajiv Gandhi Institute of Petroleum Technology, Bengaluru campus, Karnataka 562157 Bengaluru, India; 2 Rajiv Gandhi Institute of Petroleum Technology, Sivasagar Campus, Assam 785697 Sivasagar, India; 3 Department of Research and Innovation, Saveetha School of Engineering, SIMATS, Chennai, 602105 Tamil Nadu India; 4 Department of Peace and Development Studies, Njala University, Bo Campus –18, Bo, Sierra Leone

**Keywords:** Hydrogen dual fuel engine,, Response surface methodology,, Principal component analysis, Energy science and technology, Engineering

## Abstract

Hydrogen has garnered significant interest as a viable renewable energy source capable of revolutionizing conventional fuel systems. This study investigates the impact of engine load and liquid fuel injection pressure variations on the operational efficiency and emission characteristics of a hydrogen-enriched dual-fuel diesel engine, utilizing Jatropha biodiesel as the pilot ignition fuel. Advanced statistical methods, including Principal Component Analysis and Response Surface Methodology, were employed to optimize engine performance while minimizing emissions. The research aimed to achieve an Optimal Balance between efficiency requirements and emission constraints. Using Response Surface analysis, optimal engine parameters were identified at an liquid fuel injection pressure of 205.593 bar and an engine load of 70.816 %, yielding a brake thermal efficiency of 24. 3625 %, a liquid fuel replacement of 73.984 %, and NOx emissions of 188.687 ppm. These findings provide essential insights for enhancing the performance and environmental sustainability of hydrogen-assisted dual-fuel combustion systems.

## Introduction

In 2015, the United Nations instituted seventeen Sustainable Development Goals (SDGs), with Goal 7 highlighting the significance of clean and accessible energy sources. International initiatives, such as the recent G20 conference, have emphasized the necessity to reduce carbon emissions and expedite the use of renewable energy^1^. India has established lofty objectives to attain energy self-sufficiency by 2047 and achieve net-zero emissions by 2070. The nation’s growing dependence on renewable energy sources, including solar, biomass, wind, and geothermal power, supported by progressive policies such as the National Policy on Biofuels, Sustainable Alternative Towards Affordable Transportation (SATAT), and the Green Hydrogen initiative, underscores its shift towards sustainability. According to the IEA World Energy Outlook 2025 shows renewable energy will control the market but renewable electricity will be up to 6% only by 2035^2^, and the forecast published in study of Goyal et al.^3^ be indicates that 75% of 1.6 billion passenger cars operating in 2040 will run on internal combustion engines or hybrid powertrains. Also Kumar and Gautam^4^ reported that Biodiesel offers benefits of reduced greenhouse gas emissions, energy security, and sustainability. The main reason for this research focuses on optimizing technology. The method converts diesel infrastructure into a low-carbon system by using a functional approach which preserves current engine designs. Which is in support to the net zero by 2050.

Hydrogen has emerged as a prominent alternative fuel owing to its remarkable energy properties, such as a high calorific value, extensive flammability range, rapid burning rate, and negligible emissions of carbon monoxide and hydrocarbons. Hydrogen can be employed in internal combustion engines via direct combustion or fuel cells. With little adjustments, both spark-ignition and compression-ignition engines may be modified to operate on hydrogen, providing a cost-efficient decarbonization solution. Diesel engines, utilized as key power sources in heavy transportation, agriculture, and industrial sectors, significantly contribute to greenhouse gas emissions. The incorporation of hydrogen into diesel engines in a dual-fuel mode (DFM) entails the co-combustion of a gaseous main fuel (hydrogen) with a liquid pilot fuel, facilitating efficient ignition^5^. Savaş et al.^6^ reported 95% accurate model fitting for nano-MgO enriched biodiesel. While still there is scope in parameters which includes non-linear effects that may give better optimum. In terms of using biodiesel Savaş et al.^7,8^ reported that biodiesel production has a significant positive impact on global warming, as the biomass plants absorb much more CO_2_ during growth than the cumulative CO_2_ produced in the rest of the life cycle.

Studies have investigated hydrogen use in dual-fuel diesel engines (HDFDE) to meet increasing energy requirements. In this regard, Estrada et al.^5^ observed a reduction in brake-specific fuel consumption of 4–31% with the use of hydrogen. Menaa et al.^9^, through soft computing analysis, identified a liquid fuel injection pressure (LFIP) of 2.7 bar as optimal for maximizing hydrogen–air mixing. Allouis et al.^10^ reported that incorporating hydrogen in dual-fuel mode led to a reduction in particulate matter emissions. Bakar et al.^11^ reported that a hydrogen flow rate of 42.8 L/min had a significant impact on engine performance. Furthermore, Barik et al.^12^ demonstrated that combining diesel–diethyl ether with hydrogen improved engine efficiency. Benbellil et al.^13^ found that enriching natural gas with hydrogen reduced unburned hydrocarbon (HC) and carbon monoxide (CO) emissions, particularly at moderate to high engine loads. Rorimpandey et al.^14^ found that combustion instability increased at lower ambient temperatures, while Bhagat et al.^15^ reported that hydrogen combustion led to higher nitrogen oxide (NOx) emissions. Another study also showed that incorporating hydrogen into diesel combustion reduced unburned hydrocarbon and carbon monoxide emissions by 25% and 30%, respectively^16^. Hydrogen-assisted dual-fuel combustion operating without exhaust gas recirculation showed lower brake-specific energy consumption^17^ and a 1–5% rise in in-cylinder pressure^18^ compared with conventional diesel engines. Deheri et al.^19^ reported that hydrogen substitution resulted in a 30% reduction in CO emissions. Derikvand et al.^20^, through experimental investigation, found that optimal engine performance was achieved at a hydrogen injection rate of 20 mg/min. Ekin et al.^21^ observed a 12% increase in NOx emissions under hydrogen dual-fuel operation as compared with diesl mode. Gholami et al.^22^ used MATLAB-based simulations to evaluate CO₂ emissions from hydrogen dual-fuel diesel engine systems. In a separate study, Gnanamoorthi and Vimalananth^23^ optimized hydrogen flow rates and identified 30 LPM as optimal for improving efficiency. Gultekin and Ciniviz^24^ concluded from experiments that hydrogen energy ratios between 5% and 20% yielded favorable combustion characteristics^25^. In an another study, Gültekin et al.^26^ reported a 40% reduction in soot emissions at a hydrogen energy ratio of 7%.Gurbuz et al.^27^ reported that hydrogen addition in hydrogen dual-fuel diesel engines (HDFDEs) led to higher HC and NOx emissions. A comparative study of combustion chamber geometries showed that a re-entrant chamber improved brake thermal efficiency (BTE) by 3.02% relative to a hemispherical design^28^. Hosseini et al.^29^ analysed the knocking behavior in hydrogen-fueled diesel engines. In a separate study, Karimi et al.^30^ developed a computational model predicting a 1.6% rise in BTE with oxygen enrichment. Khatri and Khatri^31^ investigated a hydrogen–biogas–diesel blend. The study indicated a substantial reductions in CO and HC emissions by 88.09% and 5.68%, respectively. A number of studies have indicated that hydrogen enrichment leads to an increase exhaust gas temperature^32^, shortening ignition delay^33^, and higher NOx emissions at higher loads^34^. Kumar et al.^35^ observed that a reduction in ignition delay with higher proportions of di-tertiary-butyl peroxide and hydrogen in the air–fuel mixture. The results indicated that a 38.66% decrease in CO emissions with 40% hydrogen substitution under dual-fuel mode^36^. Kumar et al.^37^ proposed a NOx prediction model for hydrogen–diesel combustion under steady operating conditions. Lalsangi et al.^38^ examined diesel–dairy scum oil methyl ester blends with hydrogen-enriched producer gas and reported a 4.01% improvement in brake thermal efficiency^39^. Experimental investigations demonstrated that hydrogen–diesel blending enhances combustion stability^40^.

Mohite et al.^41^ applied response surface methodology (RSM) to optimize the operating parameters of a HDFDE. Based on the results, the optimized values on which the engine should be operated were found to be compression ratio of 17.73, pilot injection timing of 26.71° BTDC and energy load of 58.96%. Studies on hydrogen–diesel emulsions reported reduction in smoke emissions^42^. Research on exhaust gas recirculation (EGR)-assisted HDFDEs showed that increasing EGR levels resulted in lowering of peak cylinder pressure^43^. Another investigation recorded an 18.6% rise in exhaust gas temperature with hydrogen addition^44^. Pinto et al.^45^ observed a notable reduction in particulate matter, CO, and CO₂ emissions. Studies on HDFDEs reported a reduction of smoke opacity with hydrogen enrichment^46^, improved brake thermal efficiency under constant-volume combustion^47^. Sateesh et al. observed that the efficiency of HDFDE increased by 6.1% as the combination of dairy scum oil methyl ester and producer gas was used^48^. Seelam et al.^49^ reported that brake-specific energy consumption (BSEC) reduced with the increase of hydrogen flow rate. Balaji and Venugopal^50^ observed that NOx emissions increases with higher hydrogen substitution levels. Similar trend is reported by a separate experimental study^51^. Tripathi et al.^52^ used modeling approaches to identify the optimal injection timing for emission reduction. Tutak et al.^53^ found that hydrogen addition accelerated CNG combustion, resulting in higher peak cylinder pressure. Venkatesh et al.^54^ developed machine-learning models to predict emissions from hydrogen dual-fuel engines. Wang et al.^55^ showed that ammonia–hydrogen blends reduced CO and N₂O emissions. Winangun et al.^56^ concluded from the experimental study that 10 LPM is the optimum hydrogen flow rate required to minimize knocking in dual-fuel operation. Overall, these studies highlight hydrogen’s potential to enhance engine efficiency and reduce emissions, although challenges such as NOx formation and combustion stability require further investigation. Table 1 and Table 2 represents the recent developments especially last 5 years in the area of HDFDE.

**Table 1:** Summary of literature review of fuel, process parameters, and operating conditions for HDFDE.

**Researcher**	**Fuel**		**Process parameter**								**Operating condition**							
	Pilot fuel	Primary fuel	Specification of engine								Load	C. R	Engine speed(rpm)	H_2_ IP (bar)	H_2_ IT	H_2_ flow rate	Pilot fuel IP (bar)	Diesel IT
			Type	No. Cyl	Bore(mm)	Stroke (mm)	R.P(kW)	Speed(rpm)	IP (bar)	Dis.Vol(L)								
Gholami et al.^22^	Diesel	H_2_	7RT-flex82T 2S	7 (In-line)	0.82m	3.375m	31,640	80					80					
Allouis et al.^10^	Diesel	H_2_+methane	4S	1	85	92		1500		0.522	2,5bar	16.5:1	1,500					
Benbellil et al. (2022)^13^	Diesel	H2+NG	ListerPetterTS1 4S, DI, DE	1	95.3	88.9	4.5	1500			var	18:01	1500					
Bouguessa et al.^16^	Diesel	BG+H2	ListerPetterTS1 4S, DI, DE	1	95.3	88.9	4.5	1500			var	18:01	1500				250	13⁰bTDC
Das et al.^18^	Biodiesel	H_2_	TV1Kirloskar, CRDI, WC4S	1	110	87.5	3.5	1500	600	0.66145								
Deheri et al.^19^	Diesel	BG+H2																
Derikvand et al.^20^	Diesel	H_2_	Audi/VW TDI	4	79.5	95.5	66	4000	0.8MPa	1.896		19.5:1	2500			30lpm		
Ekin et al.^21^	Diesel	NG	DI, DE	1	137.2	165.1	74.6	2100		2.44	25%	16.25:1	910					
Gholami et al.^22^	Diesel	H_2_	7RT-flex82T,2S	7 (In-line)	0.82m	3.375m	31,640	80	19.02				80					
Gnanamoorthi and Vimalananth^23^	Diesel	H_2_	CRDI Engine 4 S	1	87.5	110	5.2	1500		0.661		17.5		2		30lpm	70Mpa	23⁰bTDC
Gürbüz et al.^27^	Diesel	H_2_	Jet Cat P80-SE, turbojet		112	290	97N	125000				2.4:1	100000	5bar		120lpm		
Halewadimath et al. (2020)^28^	Diesel	H_2_ +PG	Kirlosker TV1,4S, WC, CI	1	87.5	110	5.2	1500		0.662	80	17:01	1500	1.5bar		8lpm		
Karimi et al.^30^	Diesel	H_2_ +PG	CRDI Engine 4 S		115	125		1500	116MPa			17.5:1						
Khatri et al.^31^	Diesel	H_2_+BG	Kirloskar 4S DE, WC	1	87.5	110	3.5	1500		0.661		17.5:1	1500			18lph		
Estrada et al. (2022)^5^	Diesel	H_2_	SK-MDF300 Sokan D. I	1	78	62.57	3.5	3000		0.296		20:01	3000, 3600					20⁰bTDC
Lalsangi et al.^39^	Biodiesel	H_2_ +PG	Kirloskar 4 S D.I D. E	1	87.5	0.111	5.2	1500			80%	17.5	1500			8lpm	230	27°bTDC
Lalsangi et al.^38^	Biodiesel	H_2_	TV1,4S, WC, DI	1	87.5	110	5.2	1500	225	0.662	80	17:01				8lpm		
Lee et al.^40^	Low S Diesel	H_2_+CNG	4S naturally aspired	1	92	94			1600	0.664		17:01	1200				100	20°bTDC
M.R. Dahake and D.N. Malkhede^17^	Diesel	H_2_	4S, CRDI D.E, WC	1	87.5	110	3.5	1500		0.661	var	12 to 18	1500	2.5bar	6ms			
Mohite et al.^41^	Diesel	BG+H_2_	4S, VCR, DI, WC	1	87.5	110	3.5	1500		0.661		17.5:1	1500				200bar	23°bTDC
Muniyappan et al. ^42^	Nano diesel	H_2_	Kirloskar, 4S, WC, CI	1														
Nag et al.^43^	Diesel	H_2_	Kirlosker TV1,4S, WC, CI	1	87.5	110	5.2	1500	270	0.661	**25, 100%**	17.5:1	1500	3bar	10ms			
Paparao et al.^44^	Biodiesel	HHO	Kirloskar TA FI, AC, DI		87.5	110	4.4	1500				17.5:1				0.75lpm		
Pinto et al.^45^	HVO/FARNESANE	H_2_	Buffalo/BFDE 10.0,4S, AC	1	86	72	7.1		19.6MPa	0.418		19:01						22⁰bTDC
Raju et al.^46^	Diesel	H_2_	Kirloskar AV-1,4S, WC	1	80	110	3.7	1500	200	0.553		16.5:1						
Ramsay et al.^47^	Diesel	H_2_			115	125		1500	0.3MPa			17.5:1						
Sateesh et al.^48^	Biodiesel	H_2_ +PG	Kirlosker TV1,4S, WC, CI	1	87.5	110	5.2	1500	230	0.66						10lpm		
Seelam et al.^49^	Diesel	H_2_	Kirloskar, CRDI WC DE	1	87.5	110	35Nm	2500/3500	100MPa				1500	2	10°aTDC			
Subramanian and Thangavel^50^	Diesel	HHO	Kirloskar AV1XL 4S, DI, AC	1	87.5	80	5.97	2200	73.6	0.481	var	17:01	1850			var	210	28⁰bTDC
Tarafdar et al.^51^	Diesel	H_2_	Kirlosker TV1,4S, WC, CI	1	87.5	110	5.2/5.9	1500/1800	205	0.661	var	17.5:1	1500	1.2bar	10°ATDC		220bar	23°bTDC
Tripathi et al.^52^	Diesel	H_2_	CI		102	116	7.4	1500	250	0.9473		19.5:1						var
Venkatesh S et al.^54^	Diesel	H_2_	Kirloskar 4S AV1XL, AC	1	87.5	80	5.97	2200								var		
Wang et al.^55^	Diesel	NH3/H2			170	195	146	1800				13.5:1						19⁰bTDC
Winangun et al.^56^	Biodiesel	H_2_	DI 800H,4S, DI, WC	1	82	78	5.22	2200	220kg/cm2	0.441		18:01	2000	1bar	0°BTDC			13°bTDC

**Table 2:** Summary of emission, performance, and combustion parameters for HDFDE

**Researcher**	**Emission**					**Performance**				**Combustion**		
	CO	CO_2_	HC	NOx	Smoke	BTE	BSEC	BSFC	EGT	PCP	HRR	ID
Allouis et al.^10^	-					-	-	-	-	-	-	-
Benbellil et al.^13^					-				-			-
Bhagat et al.^15^	-		-	-	-	-	-	-	-	-	-	-
Bouguessa et al.^16^								-		-		-
Dahake and Malkhede^17^							-		-		-	-
Das et al.^18^					-			-				
Derikvand et al.^20^	-		-		-	-	-		-	-	-	
Ekin et al.^21^					-	-	-		-	-	-	-
Estrada et al.^5^					-		-	-		-	-	-
Gholami et al.^22^	-		-	-	-	-	-		-	-	-	-
Gnanamoorthi and Thangavel^23^								-				-
Gürbüz et al.^27^					-	-	-	-			-	-
Halewadimath et al.^28^		-			-		-	-	-			
Hosseini et al.^29^	-	-	-	-	-	-	-	-	-			
Karimi et al.^30^		-						-	-		-	-
Khatri et al.^31^					-						-	-
Mohite et al.^57^					-		-	-	-		-	-
Muniyappan et al.^42^								-	-	-	-	-
Nag et al.^43^	-	-		-	-	-	-	-	-		-	
Paparao et al.^44^		-					-	-		-	-	-
Pinto et al.^45^						-	-	-				-
Raju et al.^46^		-					-				-	
Ramsay et al.^47^							-	-	-	-	-	
Sateesh et al.^48^		-					-	-	-	-	-	-
Seelam et al.^49^	-							-				
Tarafdar et al.^51^	-	-			-				-	-	-	-
Tripathi et al.^52^		-	-			-	-	-				
Wang et al.^55^		-				-	-	-	-			
Winangun et al.^56^	-	-	-	-	-	-	-	-	-			-

Represents reduction in the emission/performance/combustion parameters.

Represents rise in the emission/performance/combustion parameters.

### Objective

The information regarding the effect of operating parameters like engine load (EL), compression ratio, liquid fuel injection pressure (LFIP), liquid fuel injection timings, split injection timing of the liquid fuel, gas flow rate, gas injection timings, engine speed on the performance of hydrogen-enriched dual-fuel diesel engines (HDFDE) remains limited. Few literatures are available on the application of response surface methodology for finding the optimized operating parameters settings in terms for improvement of performance and emission. This study aims to examine the effects of LFIP and EL on HDFDE performance. Additionally, response surface methodology and principal component analysis were employed to provide deeper insights into the findings. The liquid fuel utilized in this investigation is Jatropha biodiesel (JBD).

## Methods and materials

### Test set up

**Fig. 1:**
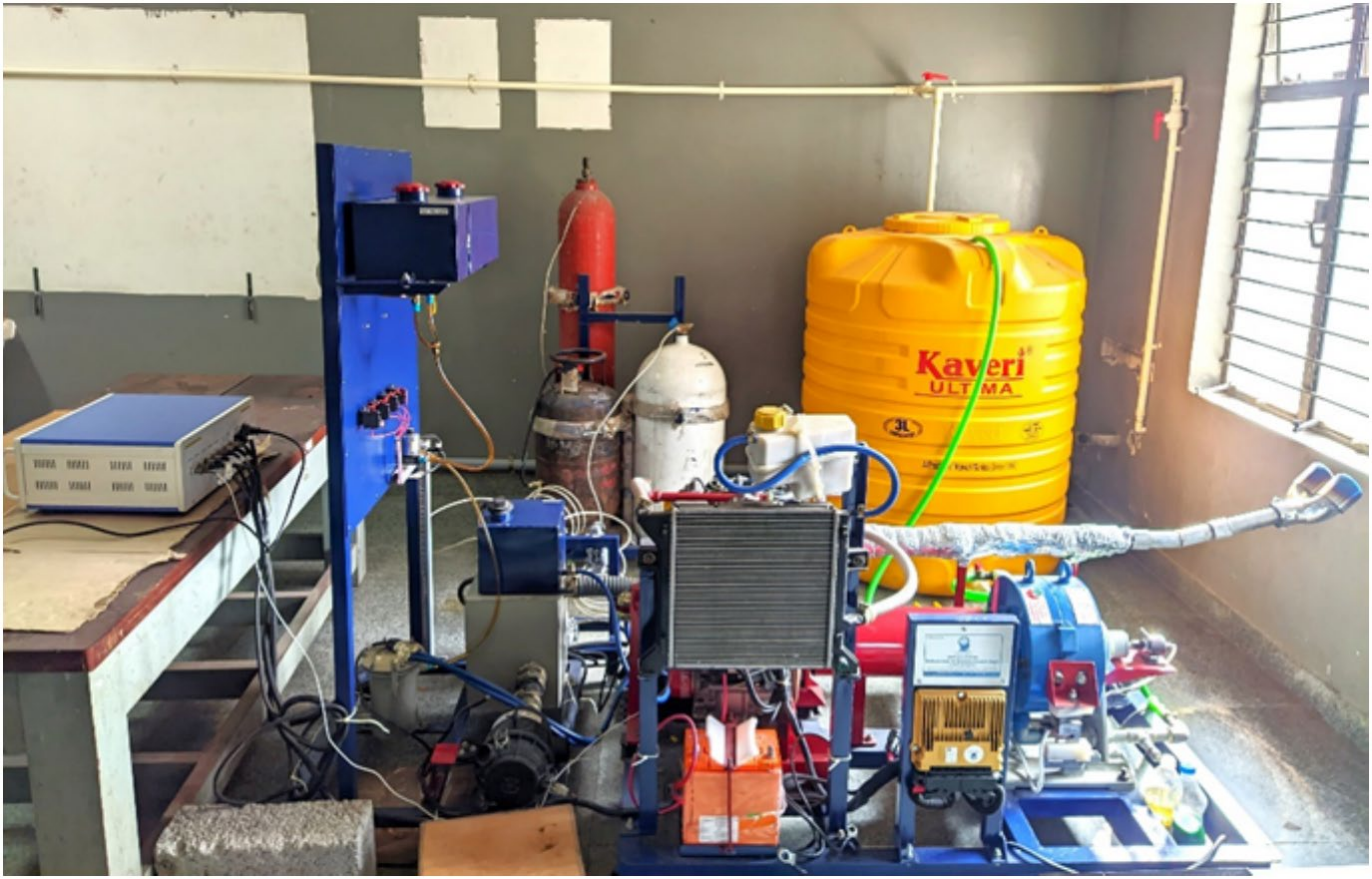
Schematic diagram of set –up.

The experimental setup features a single-cylinder, water-cooled, dual-fuel diesel engine. The primary test engine is a Mahindra and Mahindra Jeeto, single-cylinder, four-stroke, water-cooled diesel engine with a displacement of 625 cc, as depicted in Fig. 1. The Enginescan software facilitates real-time data acquisition, while flame arrestors are incorporated to enhance the safe utilization of hydrogen fuel. The engine is coupled with a water-cooled eddy current dynamometer to regulate the applied load. Additionally, a strain gauge load cell is integrated for precise load measurement. The injector operating pressure for the liquid fuel can be adjusted within the range of 180 to 220 bar. The injector is equipped with six nozzle holes, each having a diameter of 145 microns. Hydrogen is supplied using an electronically controlled secondary fuel injection system integrated with common-rail diesel injection technology and governed by the engine’s ECU software algorithms. The setup includes two independent fuel lines: one for the liquid fuel and the other for hydrogen. The hydrogen cylinder pressure is maintained at approximately 200 bar, whereas the hydrogen injector operating pressure is about 2 bar.

### Test fuel

**Fig. 2:**
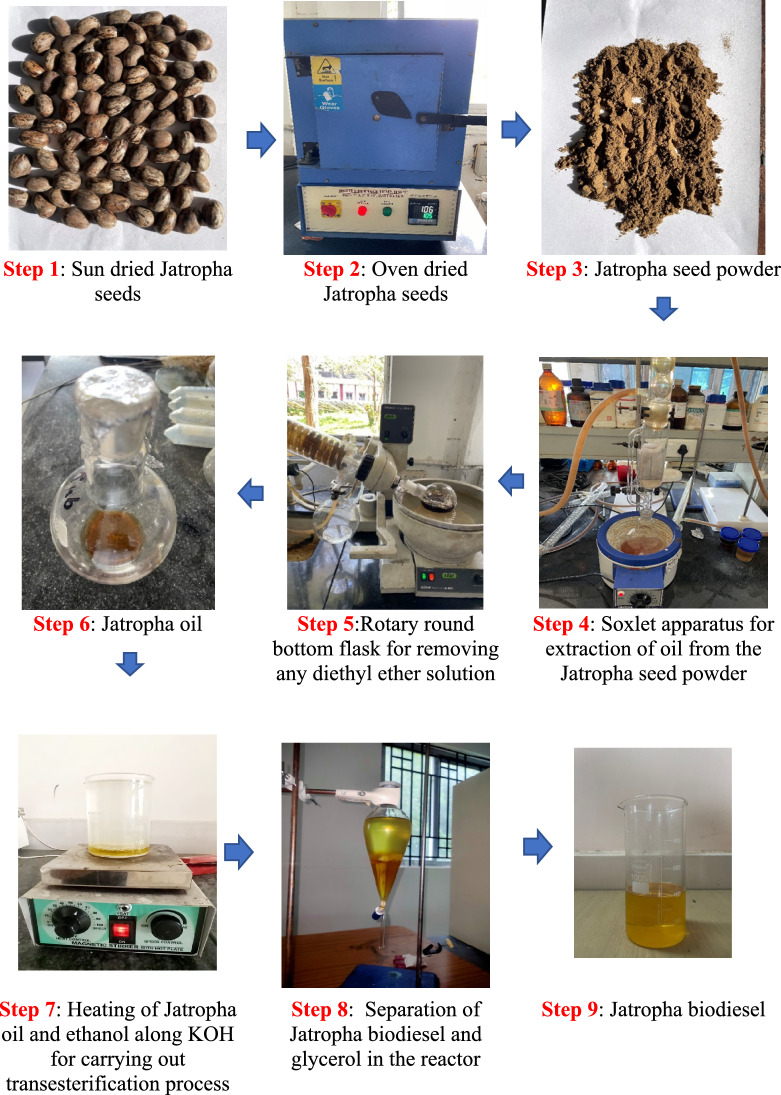
Jatropha biodiesel production process.

Hydrogen fuel, with a purity of 99%, is supplied commercially. The pilot fuel used in this study is Jatropha biodiesel. Locally available Jatropha seeds are first sun-dried, and then oven dried at 105°C. Then, oven dried Jatropha seeds were mechanically crushed to a powder form. Oil extraction from dried Jatropha seed powder is done with the help of a Soxlet Apparatus using solvents such as 10% Methylene Chloride in n-Hexane solution and 15% Diethyl Ether. Soxlet apparatus operated at 45°C for 48 hrs. The crude Jatropha oil solution is then transferred to the rotary evaporator to remove the dissolved diethyl ether present in oil. The extracted oil is then processed with ethanol in a 1:6 molar ratio in the presence of a potassium hydroxide (KOH) catalyst at 55°C. This process is called transesterification process. The reaction yields glycerol and JBD, which are subsequently separated. The entire process of JBD process is depicted in Fig. 2. The fuel properties are summarized in Table 3.

**Table 3:** Fuel properties.

Property	Hydrogen	Jatropha biodiesel
Density (kg/m^3^)	0.083	850
Cetane number	-	55
Flashpoint ( ◦C)	-	130
Fire point ( ◦C)	-	140
Calorific value (MJ/kg)	120	40.36

### Experimental matrix

Experimental conditions, including variations in operational parameters, are detailed in the experimental matrix (Table 4). During experiments, the engine is run with diesel under diesel mode (DM ) and data are recorded by the data acquisition device connected to different sensors mounted on the engine for measuring liquid fuel pressure, liquid fuel injector timing, gas pressure, gas flow rate and rpm of the engine. The diesel is drained off and fuel tank is refilled with jatropha biodiesel. Then, the engine in initially run with jatropha biodiesel. Then, hydrogen valve is slowly open till the rpm becomes steady. Then, data are recorded for dual-fuel mode (DFM). Engine tests were conducted at five EL levels (20%, 40%, 60%, 80%, and 100%) for both DM and DFM at an LFIP of 23° BTDC and an engine speed of 1500 rpm. DM refers to running the engine with diesel at the standard setting of 23 ° bTDC for LFIT and 200 bar for LFIP. For DFM, LFIP was varied at three levels (180 bar, 200 bar, and 220 bar). Initially, the engine was tested under DM according to the experimental matrix, and performance, combustion, and emission data were recorded. In DM, only diesel The tests were then repeated under DFM with hydrogen injection, and the corresponding data were collected.

**Table 4:** Experimental matrix.

Mode	EL (%)	C.R	LFIT (° bTDC)	Engine Speed(rpm)	LFIP (bar)
Diesel	20,40,60,	17.5:1	23	1500	200
Dual fuel	80,100				180 200 220

### Uncertainty analysis

**Fig. 3:**
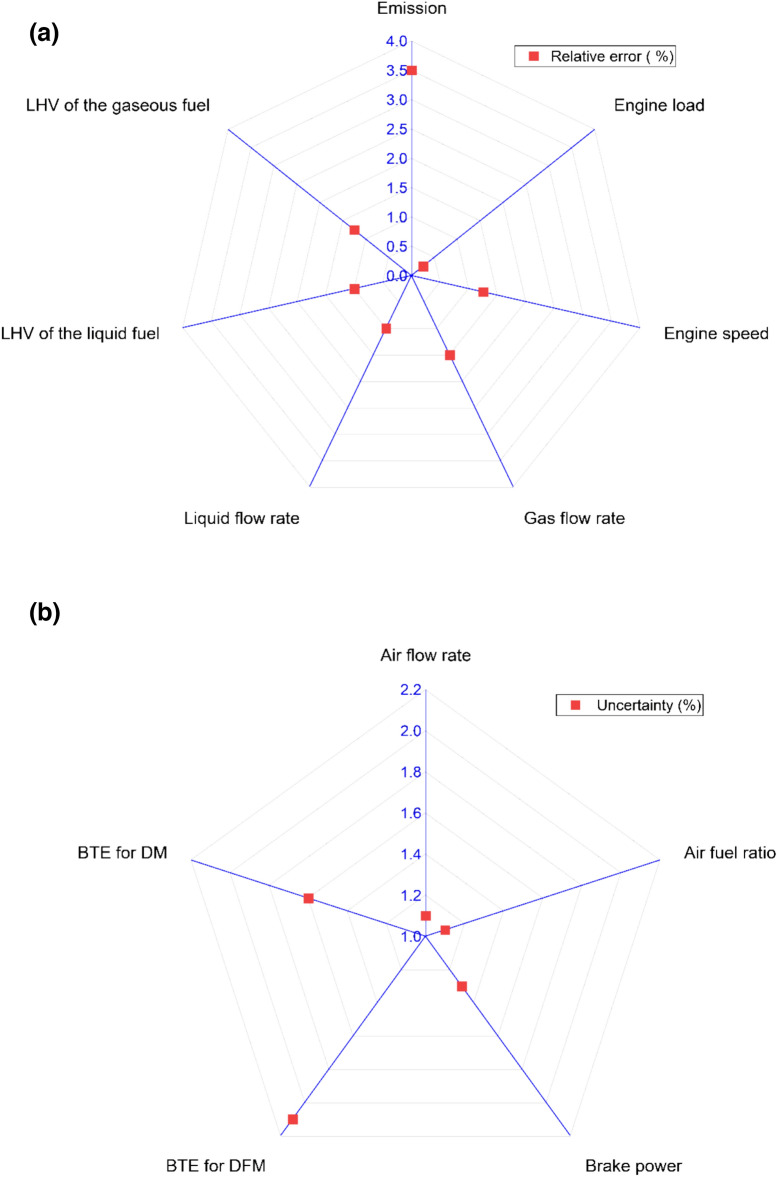
(**a**) Relative errors (**b**) Uncertainty analysis.

Uncertainty analysis plays a vital role in experimental research by quantifying potential measurement errors and variations, ensuring the reliability of results. Uncertainty analysis in an experimental study estimates the range with which true value lies. Uncertainty analysis in depends on number of factors like conditions of the measuring equipments, calibration methods, operator skills, environmental condition, instrumental error, sample size, etc. The relative errors for this study were considered from the manual and some were assumed as given in the Fig. 3(a). Using perturbation techniques, the uncertainty for the air fuel ratio, air flow rate, brake power, brake thermal efficiency for diesel mode and dual fuel node is depicted in Fig. 3(b) which demonstrates precision in experimental measurements as reported by Şener et al.^58^.

The formula for calculation of uncertainty analysis is as follows:

${\mathrm{x}}_{1}, {\mathrm{x}}_{2}, {\mathrm{x}}_{3},\dots \dots {\mathrm{x}}_{\mathrm{n}}$ are the independent parameters and A being dependant parameter which is a function of the independent parameter (as given in Eq. 1).

1 $${\mathrm{A}}_{\mathrm{n}}=\text{A }({\mathrm{x}}_{1}, {\mathrm{x}}_{2}, {\mathrm{x}}_{3},\dots \dots {\mathrm{x}}_{\mathrm{n}})$$

Then,

2 $${\Delta A} = \left[ {\left( {\frac{{\partial {\mathrm{A}}}}{{\partial x_{1} }}{\Delta A}_{1} } \right)^{2} + { }\left( {\frac{{\partial {\mathrm{A}}}}{{\partial x_{2} }}{\Delta A}_{2} } \right)^{2} + \ldots \ldots ..\left( {\frac{{\partial {\mathrm{A}}}}{{\partial x_{n} }}{\Delta A}_{{\mathrm{n}}} } \right)^{2} } \right]^{1/2}$$

where ΔA represents the uncertainty (as given in Eq. 2) generated due to uncertainties of the independent parameters ($\Delta {\mathrm{A}}_{1},\Delta {\mathrm{A}}_{2},\dots \dots .\Delta {\mathrm{A}}_{\mathrm{n}}$).

## Results and discussion

### Experimental analysis

**Fig. 4:**
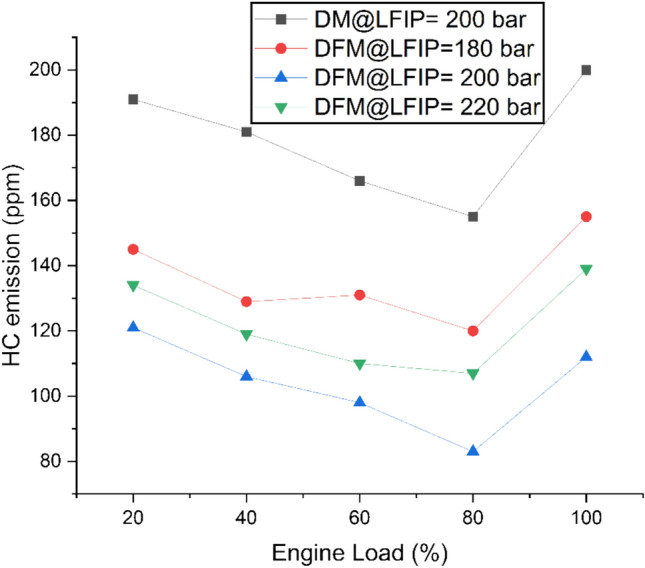
Variation of HC emissions at different ELs and LFIPs for both DM and DFM.

Fig. 4 depicts the variation of HC emissions at different ELs and LFIPs for both DM and DFM. The HC emission initially decreases, then rises to its peak under full EL conditions under DM. The same characteristics was observed at different test cases of LFIPs under DFM. The operation of the test engine under DFM results lower emission of HC. This is due to H_2_ addition increases local flame speed and diffusivity which enable more complete oxidation of hydrocarbon before freezing of reactions in expansion stroke^59^. While gaseous fuel avoid the wall wetting and diesel fuel quantity also smaller than DM which results in reduced liquid film formation and associated HC emissions^60,61^. In terms of load, at low Els, poor atomization, short spray, and small injection mass result in less heat release and a local temperature drop, which results in high HC emissions. Meanwhile, at high load, a dense spray cone, rich fuel packets, short oxidation time, and local O_2_ depletion near the spray result in higher HC emissions^62,63^. The oxygen present in JBD also contributes in lowering of the HC under DFM. The HC emission decreases initially and then increases with the LFIP variation from 180 bar to 200 bar then to 220 bar respectively. With respect to DM, an average decrease of 24%, 42% and 32% in HC emission was observed at LFIP of 180 bar, 200 bar and 220 bar, respectively for DFM. Near 200 bar and 70 to 80% Els, the droplet size may be small enough for good evaporation but not so small that fuel penetration is reduced extensively. This occurs without excessive wall impingement, which improves spatial fuel distribution and results in better charge consumption, oxidation of the charge, and overall lower HC emissions^64,65^.

**Fig. 5:**
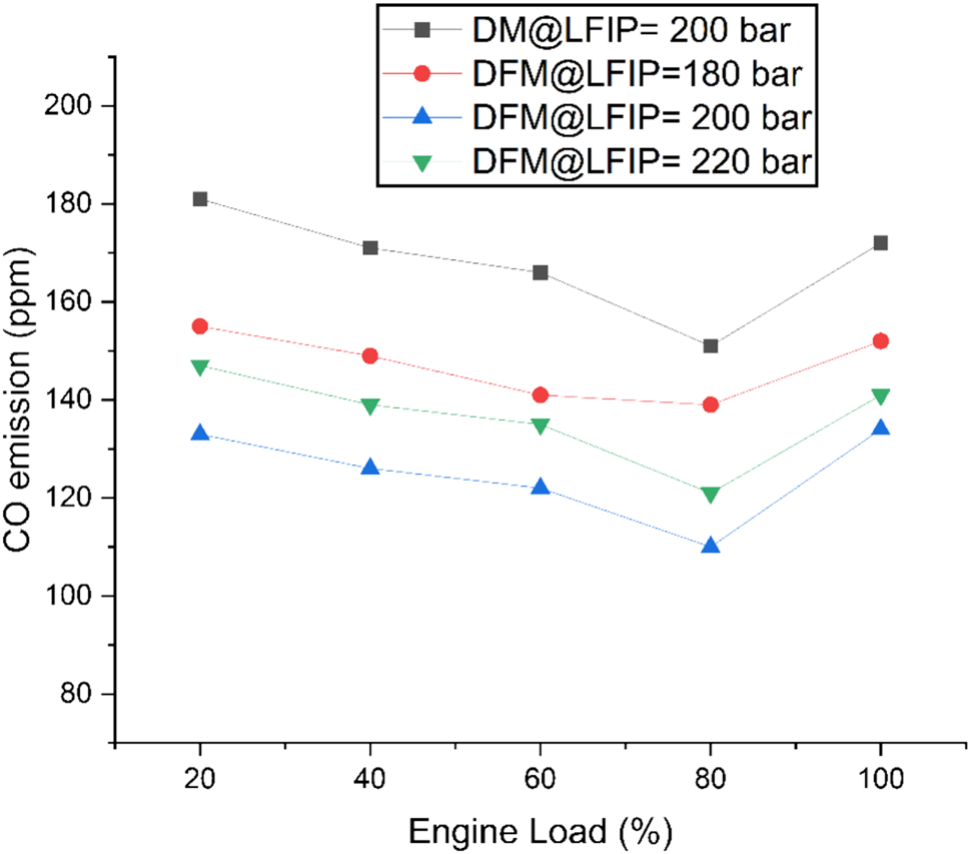
Variation of CO emissions at different ELs and LFIPs for both DM and DFM.

Fig. 5 represents variation of CO emissions at different ELs and LFIPs for both DM and DFM. In DM, the emission initially decreases and then increases with the variation of EL. The same trend of CO emission was observed under DFM. The operation of the engine under DFM lowers CO emission in comparison to DM. This is due to decrease in C/H ratio with the addition of hydrogen. The port premixed injection of hydrogen and its fast flame spread and ability to burn efficiently even in lean mixtures produce high combustion temperatures which result in better spatial heat distribution that boosts CO to CO₂ oxidation rates^66,67^. As compared to DM, an average drop of 12%, 26% and 18% in CO emission was observed at LFIP of 180bar, 200bar and 220bar, respectively under DFM. Another reason behind this may be expanded radical pool containing H and OH species enhances the CO reduction process which makes hydrogen an efficient agent for dual fuel applications to create more clean combustion technology. While it causes pre-ignition and flashback problems when the system operates at high loads which necessitates the optimization of dual fuel operating strategy^68,69^.

**Fig. 6:**
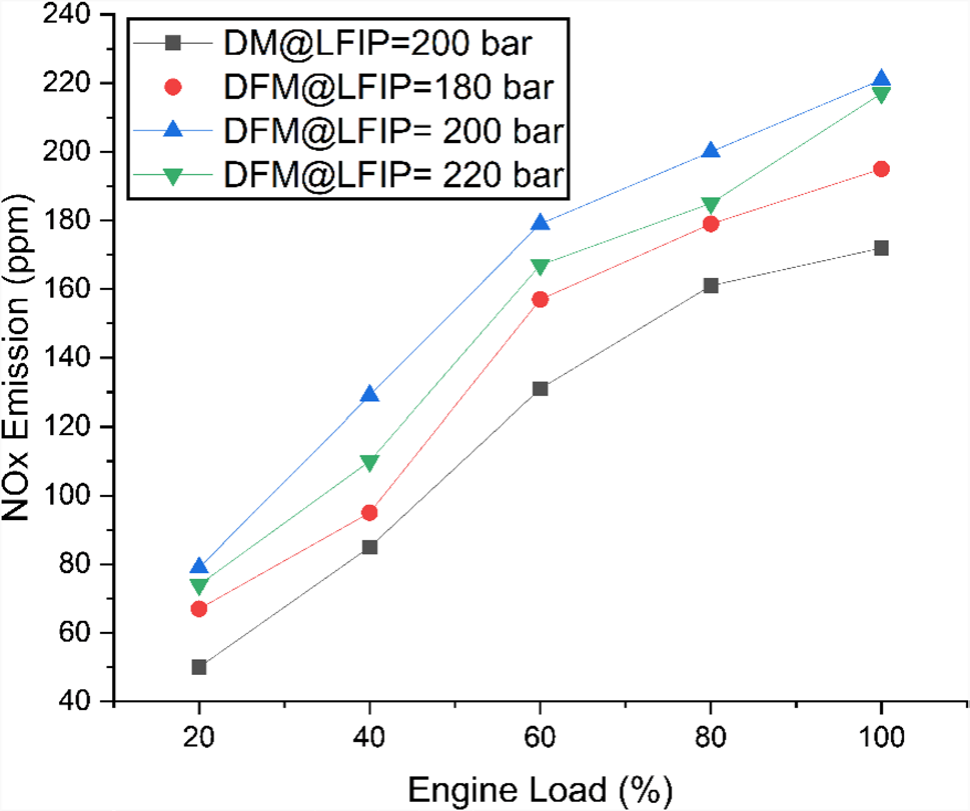
Variation of NOx at different ELs and LFIPs for both DM and DFM.

Fig. 6 represents the NOx emission with the variation of EL and LFIP for both DM and DFM. The extended ignition delay period in DFM results in higher NOx emissions because it extends the time required for fuel-air mixture blending which leads to higher combustion chamber temperatures. The temperature increase enables the Zeldovich mechanism to produce thermal NOx through its reaction pathway. The combustion reactivity of JBD increases because it contains unsaturated compounds and built-in oxygen which produces higher cylinder temperatures that result in NOx formation. The dual fuel system which operates with hydrogen and biodiesel fuel produces elevated NOx emissions because engine load and biodiesel injection pressure create elevated peak combustion temperatures which result in accelerated reaction rates because hydrogen flames spread as load increases^70,71^. There is an average increase of NOx emission by 16%, 35% and 26% for LFIP of 180 bar, 200 bar and 220 bar, respectively as compared to DM.

**Fig. 7:**
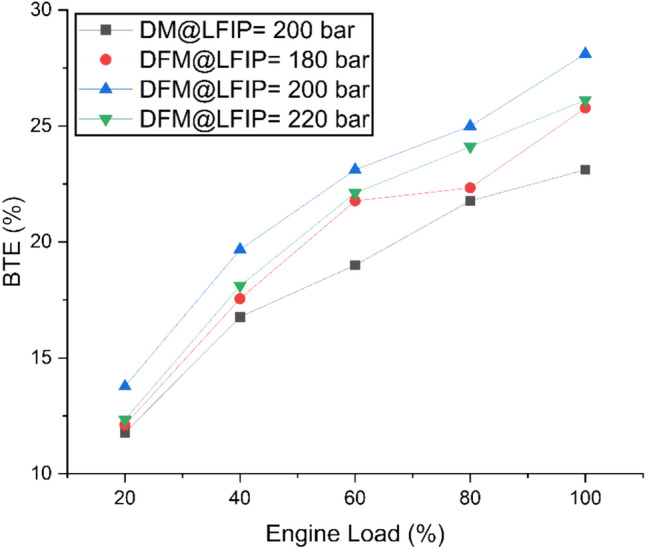
Variation of BTE at different ELs and LFIPs for both DM and DFM.

Fig. 7 represents variation of BTE at different ELs and LFIPs for both DM and DFM. The BTE rises with increase in EL for both DM and DFM. However, BTE for the DM is lower compared to when the engine is running on DFM. This may be due to the high calorific value, flame speed and wider flammability range of hydrogen. Further, in dual-fuel operation, the gaseous nature and high diffusivity of hydrogen promote superior air–fuel mixing, leading to a more homogeneous charge^72^. The system produces better uniformity in local equivalence ratio which leads to faster flame spread and increased combustion temperatures inside the cylinder. Which leads to shorter ignition times and improved brake thermal efficiency when operating with biodiesel fuel as the only fuel source^57,73^. Another factor that contributes synergistically in the improvement of the combustion efficiency is the presence of oxygen in JBD. BTE was observed to be highest when the LFIP was 200bar. The DFM test revealed that an increase in BTEs by 7%, 19% and 11% for LFIP of 180bar, 200bar and 220bar, respectively as compared to DM. This may due to the fact as LFIP increased to 200 bar, the fuel pattern changes to a finer spray resulting in the formation of smaller droplets and enhances air fuel reactivity. Thus, improving the combustion process and hence, resulting in a better efficiency. Further increase of LFIP to 220 bar may result in the increase of spray penetration length which causes the impingement of the injection spray pattern on the piston wall. This lowers the fuel air ratio which causes in lowering of the BTE.

**Fig. 8:**
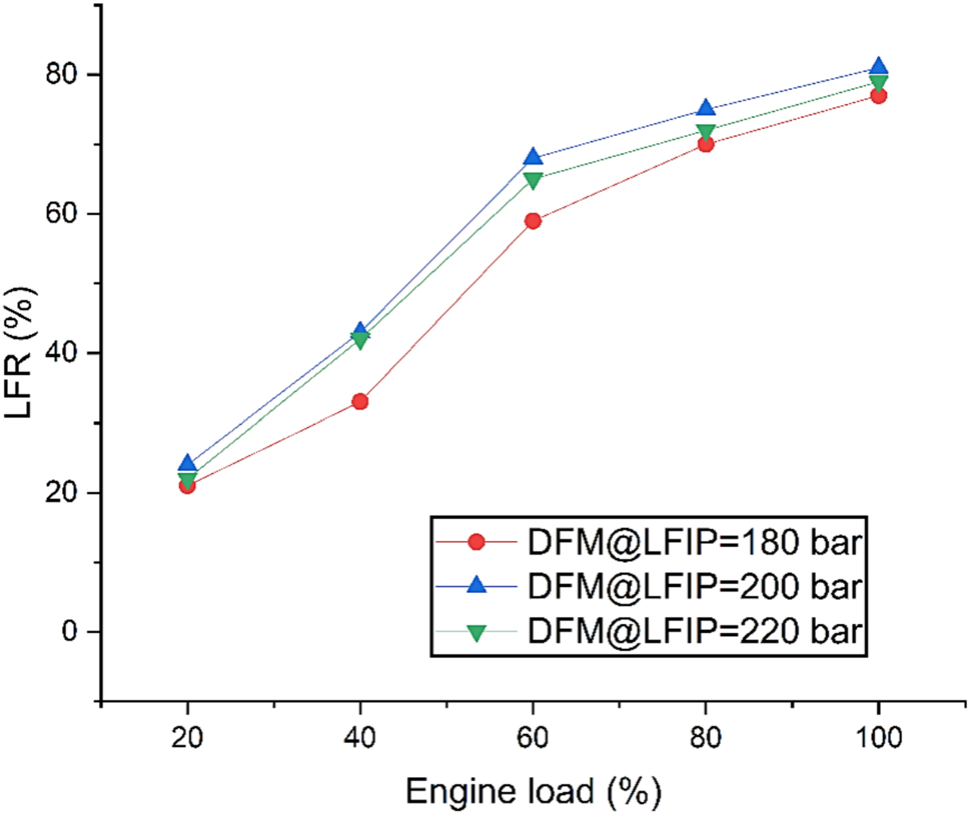
Variation of LFR at different ELs and LFIPs for both DM and DFM.

Fig. 8 shows the variation of LFR at different ELs and LFIPs both DM and DFM. It is seen that the LFR increases with the operation of the DFM at higher engine loads, in agreement with previous studies on hydrogen–biodiesel dual-fuel engines^74^. For all tested Liquid LFIP, the highest LFR values occurred under full engine load conditions, consistent with optimization findings that link high load and mid-range LFIP to maximum hydrogen substitution^71^. The maximum LFR of 81% was achieved at an LFIP of 200 bar, comparable to reported optima of ~80–85% . At LFIP settings of 180 bar and 220 bar, LFR decreased by 4% and 1%, respectively, relative to 200 bar, reflecting the sensitivity of LFR to injection pressure deviations and needs to be optimized.

**Fig. 9:**
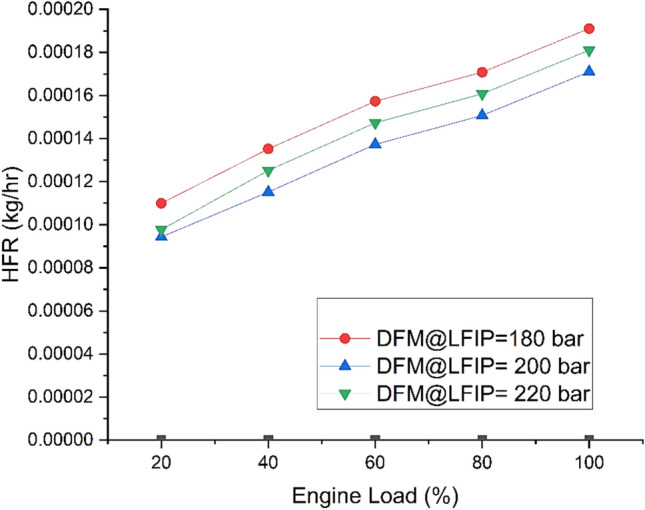
Variation of HFR at different ELs and LFIPs for both DM and DFM.

Fig. 9 depicts the variation of HFR at different ELs and LFIPs both DM and DFM. HFR increases with the operation of DFM at high loads. This may be due to the high calorific value and high flammability of hydrogen along with oxygenated pilot fuel in form of JBD which resulted in a higher heat release in the premixed stage. The maximum values for HFR for all three cases of injection pressure was observed at full EL condition. The average maximum value for the HFR was observed to be higher by 14% and 6.5% at LFIP of 180 bar and 220 bar, respectively as compared to 200 bar under DFM.

**Fig. 10:**
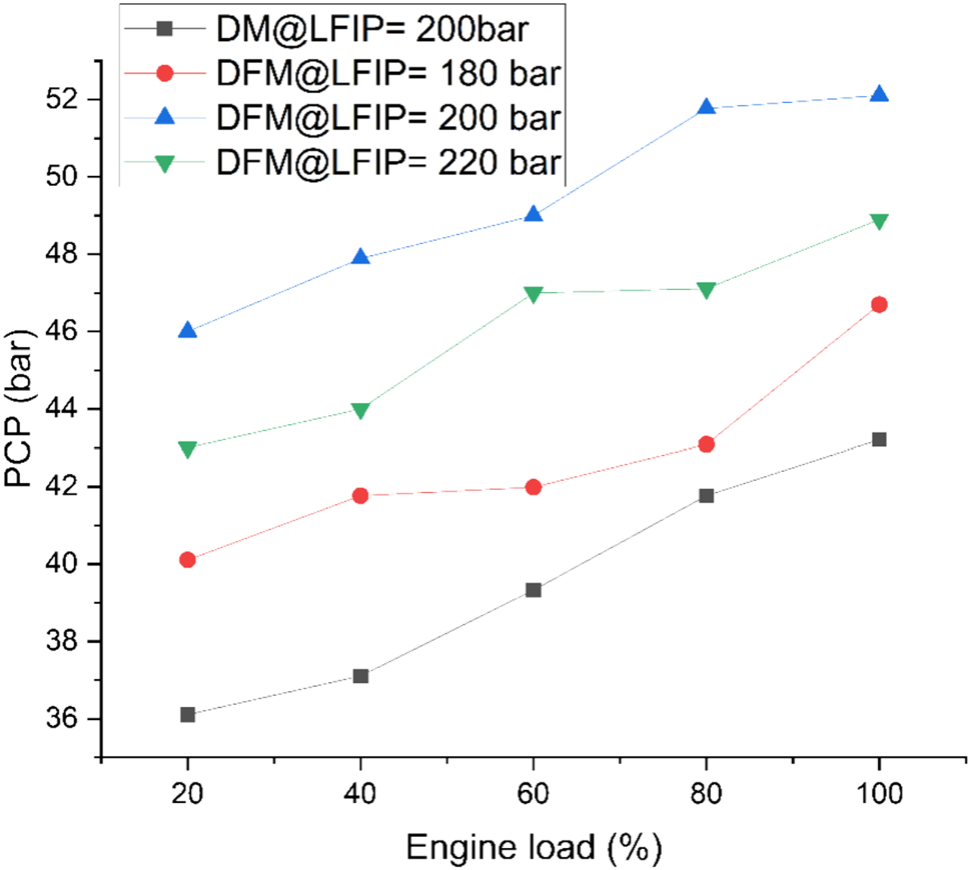
Variation of PCP at different ELs and LFIPs for both DM and DFM.

Fig. 10 indicates the variation of PCP at different ELs and LFIPs for both DM and DFM. The PCP increases with operation at higher loads for both DM and DFM. PCP for the DM is lower as compared to DFM. This may be attributed to hydrogen’s higher flammability and faster combustion rate compared to diesel fuel. The addition of hydrogen to JBD leads prolongs the ID which results in the combustion of the major part of the charge in the premixed stage and thereby, resulting a higher turbulence inside the combustion chamber and excessive pressure. The PCP increases and then decreases as the LFIP was varied from 180 bar to 200 bar then to 220 bar respectively. Analyses shows PCP to be highest when the LFIP was 200 bar. During DFM, there is an average increase in PCP by 8%, 25% and 16% at LFIP of 180 bar, 200 bar and 220 bar, respectively in comparison to DM.

**Fig. 11:**
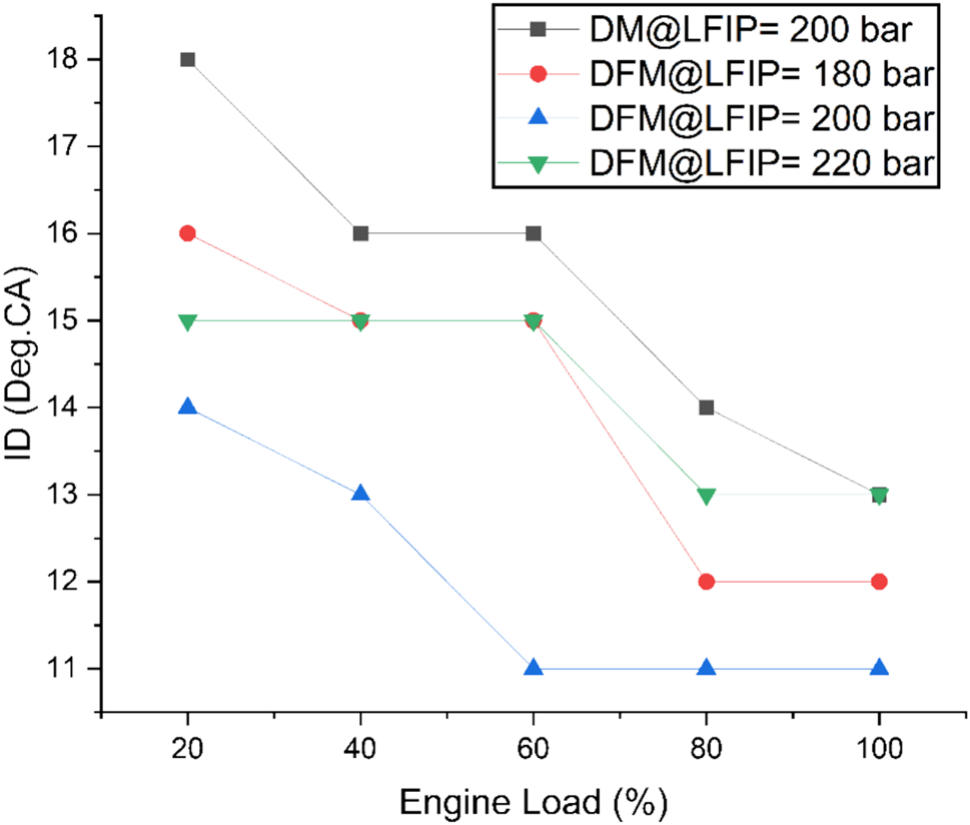
Variation of ID at different ELs and LFIPs for both DM and DFM.

Fig. 11 exhibits the variation of ID at different ELs and LFIPs for both DM and DFM. With increasing EL, the ID decreases for both DM and DFM. However, hydrogen has a low Cetane number and high self-ignition temperature as compared to diesel fuel. The addition of hydrogen to JBD prolongs the preignition reaction and thus, resulting in longer ID under DFM as compared to DM. It was observed that the maximum value of ID was observed at LFIP of 180bar and 20% EL condition on operation of the engine under DFM. Whereas ID was found to be minimum at full EL and LFIP of 200bar. The operation of the engine under DFM resulted in an average decrease of ID by 9%, 22% and 8% at LFIP of 180bar, 200bar and 220bar, respectively as compared to DM.

### Multi-method statistical optimization

#### correlation analysis

**Fig. 12:**
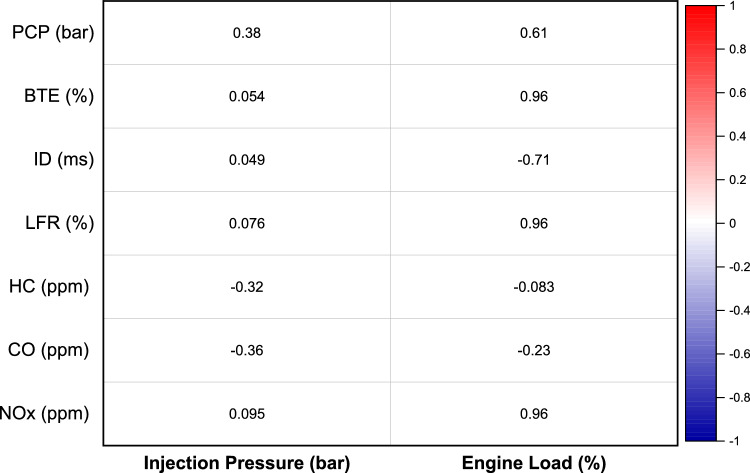
Correlation plot for operating points vs performance, and emissions.

The correlation analysis reveals the relationships between input parameters (“Injection Pressure” and “EL”) and various output performance and emission parameters of a dual-fuel engine as depicted in Fig. 12. The results indicate that EL has a substantial influence on most output variables, with strong positive correlations observed with Brake Thermal Efficiency (BTE) (r = 0.96), Liquid Fuel Replacement (r = 0.96), and NOx Emissions (r = 0.96). These results suggest that increasing EL significantly enhances thermal efficiency and fuel replacement; however, it also leads to a corresponding rise in NOx emissions. In contrast, Ignition Delay exhibits a strong negative correlation with EL (r = -0.71), indicating that higher EL reduces ignition delay. Injection Pressure shows a relatively moderate impact on emissions, particularly on HC and CO emissions, with moderate negative correlations (r = -0.32 and r = -0.36, respectively). The results indicate that higher injection pressure plays a role in lowering emissions; however, its influence on essential engine performance metrics like brake thermal efficiency (BTE) and peak cylinder pressure is relatively minor. In general, the findings emphasize that fine-tuning the energy level (EL) is critical for improving efficiency and maximizing fuel substitution. Nonetheless, this improvement is accompanied by a rise in NOx emissions, which raises environmental concerns. On the other hand, adjusting injection pressure proves effective in reducing hydrocarbon (HC) and carbon monoxide (CO) emissions. Consequently, a well-balanced strategy is necessary to enhance engine performance while minimizing pollutant emissions, carefully considering the trade-offs identified in these interactions. The research used correlation analysis to evaluate linear relationships between engine load and injection pressure as input variables and BTE and NOx and HC as output variables which produced first insights about performance and emission-controlling factors. While PCA to confirms the initial reduced factor linear relationships discovered through correlation analysis to improve RSM optimization results for sustainable engine development as given in next section.

#### Principal component analysis (PCA)

**Fig. 13:**
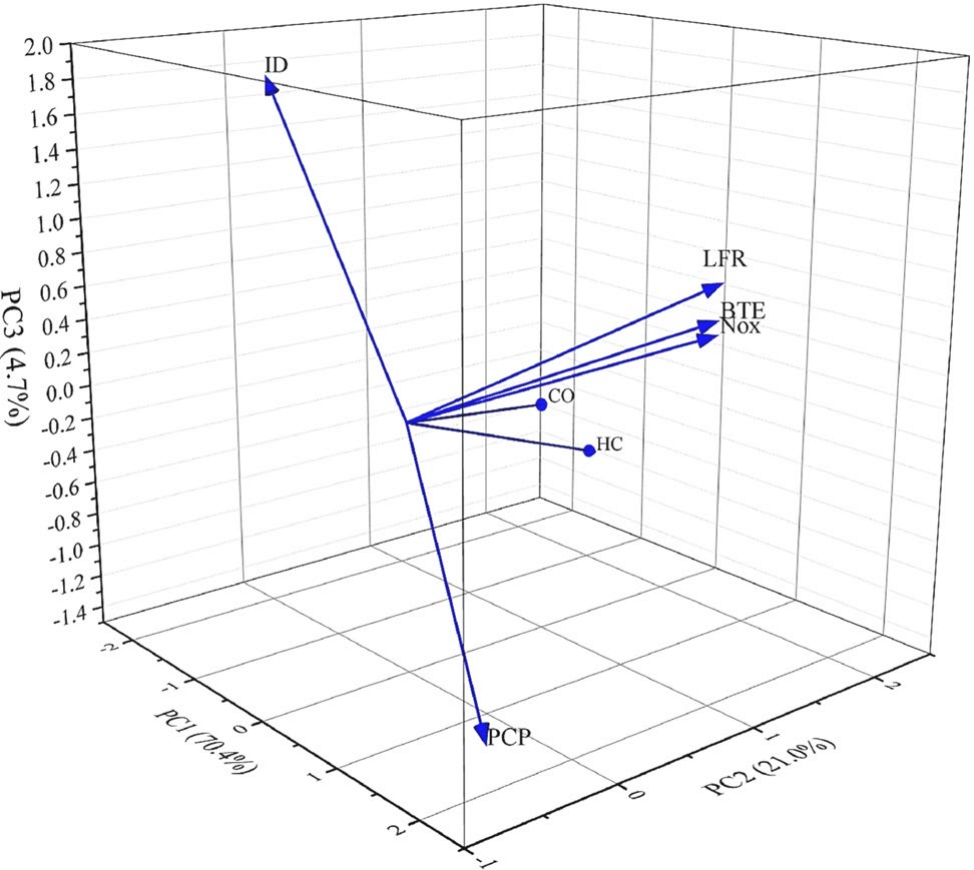
PCA biplot for performance, and emissions parameters.

Principal Component Analysis (PCA) was conducted after the correlation evaluation to provide a more profound understanding of the complex relationships between engine performance indicators and emissions, as seen in Fig. 13. This method was utilized to reduce the high-dimensional information and identify the most significant characteristics influencing engine efficiency and emissions. PCA simplifies dimensional complexity, facilitating a clearer grasp of the interrelationships among variables like Brake Thermal Efficiency (BTE), Liquid Fuel Replacement (LFR), emissions (NOx, CO, HC), Peak Cylinder Pressure (PCP), and Ignition Delay (ID). This, thus, facilitates the development of effective methods that both enhance performance and reduce environmental impact. The PCA biplot provided essential insights into the relationships among these crucial factors. The first principal component (PC1) accounts for 70.4% of the data variation, while the second principal component (PC2) represents 21.9%, collectively capturing 92.3% of the overall variance. The third principal component (PC3) accounted for an extra 4.7% of variability, indicating that the key trends in the dataset were mostly captured by the first two components.

The biplot analysis revealed a robust positive association among brake thermal efficiency (BTE), liquid fuel replacement (LFR), and nitrogen oxide (NOx) emissions, shown by the analogous direction of their vectors. This result demonstrates that improvements in engine efficiency and more replacement of liquid fuel correlate with elevated NOx emissions, a recognized trade-off in combustion systems. Conversely, peak cylinder pressure (PCP) and ignition delay (ID) exhibited an inverse association, as their vectors oriented in opposing directions. The results indicate that increased cylinder pressure decreases ignition delay, underscoring the possibility of modifying ignition time by cylinder pressure adjustment. Moreover, carbon monoxide (CO) and hydrocarbon (HC) emissions exhibited a positive correlation, as indicated by the closeness of their vectors. Nonetheless, these emissions had a tenuous correlation with BTE and LFR, suggesting that fluctuations in efficiency and fuel substitution do not directly influence their generation. The unique positioning of PCP and ID concerning efficiency-related elements highlights that, despite enhancements in fuel economy and replacement levels, meticulous regulation of combustion characteristics, including pressure and ignition time, is essential to avert adverse side effects.

The PCA highlighted significant trade-offs between engine efficiency and emissions, notably the simultaneous increase in NOx emissions and enhancements in thermal efficiency and fuel substitution. These insights underscore the necessity of implementing tailored optimization measures to reconcile efficiency and emissions, particularly focusing on minimizing NOx while maintaining high brake thermal efficiency (BTE). The results provide a crucial basis for formulating sophisticated control algorithms to enhance dual-fuel engine performance and reduce environmental impact.

### Optimization of response surface models using a gaussian process model

**Fig. 14:**
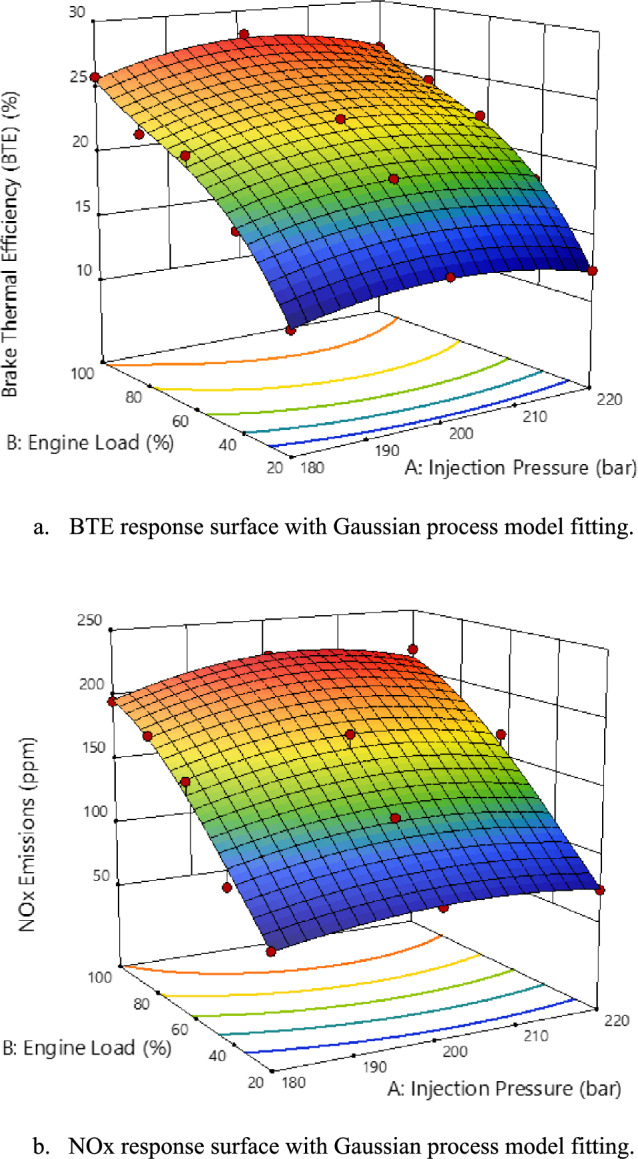
EL and Injection Pressure RSM Model Fit.a.BTE response surface with Gaussian process model fitting.b.NOx response surface with Gaussian process model fitting.

The engine parameters optimization for load and injection pressure was conducted to achieve a desirable balance between BTE and NOx emissions. The Gaussian process model^75^ response surfaces methodology^41^ for each are visualized in the 3D plots, revealing the relationship between these parameters and the performance and emission outputs. The BTE response surface shows a clear increase in efficiency with rising EL and moderate increases in injection pressure. As shown in the BTE plot Fig. 14. (a), maximum efficiency, approximately 28.11%, is observed at higher ELs combined with moderate-to-high injection pressures. Lower loads and injection pressures result in a drop in BTE, reaching a minimum value of around 12.11%. These findings indicate that operating the engine at a higher load with optimized injection pressure can improve thermal efficiency, a crucial factor for performance optimization. The NOx emissions plot as represented in Fig. 14 (b), reveals a trade-off between efficiency and emissions. NOx levels tend to increase with both higher EL and injection pressure, reaching a peak of about 221 ppm. At lower loads and injection pressures, NOx emissions decrease, with a minimum value of around 67 ppm. This relationship aligns with the known trade-off in internal combustion engines, where increasing efficiency (through higher loads and pressures) often leads to higher NOx production due to higher combustion temperatures.

To ensure the reliability of the optimization results, the gaussian process model was evaluated using several statistical performance indicators as presented in Table 5. The model demonstrates high predictive accuracy, with cross-validated R2 values ranging from 0.8820 to 0.9952. These values are well within the highly acceptable range for internal combustion engine studies. The model achieved its best performance when smoothing and noise parameters were set to optimize between model flexibility and experimental data precision because the overall noise values remained low. The results meet all scientific requirements for rigor which allows us to apply them as dependable data for RSM-based optimization analysis and further used in optimization.

**Table 5:** Gaussian process model statistics for DFM engine performance and emissions.

	PCP	BTE	ID	LFR	HC	CO	NOx
**Smoothing Parameter**	4.76	19.31	184.21	2.68	0.7000	23.48	2.40
**Noise Parameter**	0.0142	0.0000	0.0000	0.0663	0.0651	1.000E-05	0.1580
**Overall Scale**	52.30	27398.60	48778.06	50.83	87.96	3.874E+05	48.64
**Overall Noise**	0.7451	0.4933	0.6853	3.37	5.73	3.87	7.68
**R Squared**	0.9724	0.9952	0.8820	0.9858	0.9937	0.9484	0.9845

The desirability function^76–78^ was applied to balance the trade-off between BTE and NOx emissions, considering that both are important for optimal engine performance and emissions compliance. However, the function’s maximum desirability was constrained below 1 to reflect this inherent trade-off, particularly between BTE and NOx levels as indicated in Table 6. The optimum solution achieved, presented in Table 7, represents a balanced setting of EL and injection pressure that maximizes BTE while keeping NOx emissions within an acceptable range, based on the response surface analysis.

$$D_{F} = (d_{1} {\kern 1pt} d_{2} \cdots d_{n} )^{\frac{1}{n}} = \left( {\prod\limits_{i = 1}^{n} {d_{i} } } \right)^{\frac{1}{n}}$$

**Table 6:** Optimization criteria table.

**Name**	**Desired goal**	**Range**	**Importance**	${D}_{F}$
A: LFIP (bar)		180-220	3	0.643092
B: EL (%)		20-100	5	0.658454
PCP (bar)		40.11-52.11	3	0.847755
BTE (%)		12.11-28.11	5	0.778133
ID (ms)		11-16	3	0.833853
LFR		21-81	5	0.856975
HC (ppm)		83-155	3	0.928247
CO (ppm)		110-155	3	0.897952
NOx (ppm)		67-221	5	0.190287

**Table 7:** Optimum solution table.

**LFIP** **(bar)**	**EL (%)**	**PCP (bar)**	**BTE (%)**	**ID (ms)**	**LFR (%)**	**HC (ppm)**	**CO (ppm)**	**NOx (ppm)**	***D***_**F**_
205.593	70.816	50.1437	24.3625	11.8913	73.984	89.0196	114.774	188.687	0.650

## Conclusion

The study highlights the critical trade-offs between engine performance and emissions in a HDFDE, emphasizing the interplay between injection pressure and EL. The pilot fuel considered for this study was Jatropha biodiesel. Experiments were carried on a dual fuel test set up. The summary of the performance, PCA and RSM analysis is as follows:

1. EL is a dominant factor influencing both thermal efficiency and emissions, with higher loads improving Brake Thermal Efficiency (BTE) and Liquid Fuel Replacement (LFR) but significantly increasing NOx emissions.
2. Injection pressure moderately reduces HC and CO emissions, demonstrating its potential for emission control, while its effect on efficiency related parameters remains limited.
3. Principal Component Analysis (PCA) confirms the strong correlation between BTE, LFR, and NOx emissions, highlighting the need for targeted strategies to balance efficiency gains with emission constraints.
4. Optimal operating conditions were determined using Gaussian Process Model Based RSM are fuel injection pressure of 205.593 bar and an EL of 70.816 %, resulting in a BTE of 24.3625 %, LFR of 73.984 %, ID 11.8913 ms, HC 89.0196 ppm and CO 114.774 ppm and NOx emissions of 188.687 ppm with cross validation model accuracy of R^2^ 0.8820 to 0.9952.

Based on the above results, a biodiesel–hydrogen dual-fuel engine can be considered a viable solution for clean power generation, provided the hydrogen is produced from renewable sources.

## Data Availability

The datasets during and/or analyzed during the current study are available from the corresponding author upon reasonable request.

## Abbreviations

ATDC: After top dead center
AC: Air cooled
BTDC: Before top dead centre
Bn: Billions
BTE: Brake thermal efficiency
CO_2_: Carbon dioxide
CO: Carbon monoxide
CNG: Compressed natural gas
CR: Compression ratio
CRDI: Common rail direct ignition
DE: Diesel engine
DFM: Dual fuel mode
DI: Direct ignition
DM: Diesel mode
EL: Engine load
EGT: Exhaust gas temperature
G20: Group of twenty
HC: Hydrocarbon
HDFDE: Hydrogen dual fuel diesel engine
HRR: Heat release rate
ID: Ignition delay
IMEP: Indicated mean effective pressure
IP: Injection pressure
IT: Injection timings
JBD: Jatropha biodiesel
LF: Liquid fuel
LFIP: Liquid fuel injection pressure
ML: Machine algorithm
NOx: Nitric oxides
PCP: Peak cylinder pressure
PG: Producer gas
Ppm: Parts per million
Rpm: Revolution per minute
SDG: Sustainable development goals
UHC: Unburned hydrocarbon
WC: Water cooled
4S: Four stroke
2S: Two stroke

## Symbols

$: Dollar
°: Degree
%: Percentage
